# Secondary Prophylaxis Among First Nations People With Acute Rheumatic Fever in Australia: An Integrative Review

**DOI:** 10.1177/10436596231191248

**Published:** 2023-08-12

**Authors:** Kerissa Govender, Amanda Müller

**Affiliations:** 1 Flinders University, Adelaide, South Australia, Australia

**Keywords:** acute rheumatic fever, rheumatic heart disease, indigenous peoples, secondary prevention

## Abstract

**Introduction:**

The prevalence of acute rheumatic fever (ARF) and rheumatic heart disease (RHD) among Australia’s First Nations populations are some of the highest in the world, accounting for 95% of the 2,244 ARF notifications between 2015 and 2019 in Australia. A key issue in treating ARF is long-term secondary prophylaxis, yet only one in five patients received treatment in 2019. This review identifies barriers to secondary prophylaxis of ARF in Australia’s First Nations people.

**Methods:**

An integrative review was undertaken utilizing PubMed, CINAHL, ProQuest, and Wiley Online. Joanna Briggs Institute critical appraisal tools were used, followed by thematic analysis.

**Results:**

The key themes uncovered included: issues with database and recall systems, patient/family characteristics, service delivery location and site, pain of injection, education (including language barriers), and patient-clinician relationship.

**Conclusions:**

A national RHD register, change in operation model, improved pain management, improved education, and need for consistent personnel is suggested.

## Background

Acute rheumatic fever (ARF) is the consequence of an autoimmune response to *A streptococcus* infections (RHD Australia, 2020). Left untreated, rheumatic fever can progress to rheumatic heart disease (RHD), an inflammatory process that can cause acute inflammation of cardiac muscle and cause chronic fibrosis of the cardiac valves (Ralph et al., 2016), which often requires surgical intervention. Screening and diagnosis of ARF can be challenging for health practitioners as there is no single test or procedure to diagnose it. Instead, it is screened and diagnosed through assessment of past medical history, physical examination, and laboratory tests (Alqanatish et al., 2019). The Jones criteria devised in 1944 outlined the manifestations of Rheumatic Fever (Alqanatish et al., 2019). In 2015, the American Heart Association made diagnostic revisions to include minor and major criteria for low, moderate, and high risk (Alqanatish et al., 2019).

Current approaches for the prevention and management of ARF and RHD are based around primordial, primary, secondary, and tertiary prevention strategies (RHD Australia, 2020). The reduction of risk factors is covered in primordial prevention and aims to look at the social determinants of health that increase the risk of infection from *A streptococcus* (Coffey et al., 2018). Primary prevention focuses on the identification and treatment of *A streptococcus* infections that cause ARF (RHD Australia, 2020). Secondary prevention (the focus of this study) encompasses secondary prophylaxis of antibiotic administration (benzathine benzylpenicillin intramuscular injections) and other strategies that control *A streptococcus* infections and subsequent progression of ARF and RHD (RHD Australia, 2020). Finally, tertiary prevention of RHD involves the treatment of the consequences of the disease process such as treatment for stroke, arrhythmias, heart failure, and valvulopathies (Ralph et al., 2016). ARF, and its subsequent progression to RHD, are considered almost completely preventable (Kang et al., 2021). ARF and RHD are often considered as diseases of poverty and disadvantage due to its prevalence in predominantly low socioeconomic settings (Kang et al., 2021). This article also identifies the importance of remote geographic location and cultural factors contributing to these diseases.

In Australian settings, ARF and RHD are predominantly found in remote and very remote settings (Katzenellenbogen et al., 2020). In the 1930s and 1940s in Australia, ARF and RHD were prevalent among the general Australian population in the large city of Melbourne (Cannon et al., 2019). However, improved living conditions, better hygiene practices, and access to penicillin-based medications and health services (RHD Australia, 2020) meant these disease processes were almost completely eradicated and ARF now rarely occurs there (Cannon et al., 2019). Today, ARF and RHD most often occur among Australia’s First Nations populations (Australian Aboriginal and Torres Strait Islander) and the prevalence is among the highest in the world (Chamberlain-Slaun et al., 2016). From 2015 to 2019, there were 2,128 notifications for ARF among First Nations people, comprising 95% of the total notifications (Australian Institute of Health and Welfare [AIHW], 2021). A notification is defined as the diagnosis of ARF, which can occur in a patient numerous times (AIHW, 2021). In 2019, the incidence of ARF was 5/100,000 for the combined populations of Queensland, Northern Territory, South Australia, and Western Australia (AIHW, 2021). However, when First Nations incidences are calculated within their own populations for 2019, the incidence of ARF was 102/100,000 (AIHW, 2021), revealing the true prevalence of this disease.

Inequity in access to primary health care services contributes to the prevalence of chronic diseases such as ARF (Davy et al., 2016; Kang et al., 2021). This is not only due to distance from health care services but also barriers such as patient communication with health care practitioners, discrimination, and cost (Davy et al., 2016; Jones et al., 2020). The gap in care is, in part, due to the socioeconomic disadvantage and health inequality that exists for First Nations communities (AIHW, 2017; Kang et al., 2021; Wiemers et al., 2018). The inequality and disadvantage are a consequence of the colonization of Australia and subsequent suppression of culture, language, and tradition that have occurred in many communities (Haynes et al., 2020). Within health care, systemic racism has resulted in an implicit bias against First Nations people (Kerrigan et al., 2021; Mayes, 2020). Research by Shirodkar (2019) suggests that up to 75% of Australians hold an implicit negative bias against Indigenous Australians. Implicit bias may occur unintentionally or unconsciously, and can have a significant impact on the experiences and outcomes of those who experience it (Quigley et al., 2020). For Australia’s First Nations people, implicit bias within health care can come in the form of stereotyping, differing quality of care, and dismissiveness of patient symptoms (Quigley et al., 2020). First Nations people are also socioeconomically disadvantaged in relation to income, housing, education, and employment (AIHW, 2011), which has a direct link to increased risk of morbidity and mortality due to limited opportunities to improve health (Mitchell et al., 2018; Wiemers et al., 2018). Furthermore, when First Nations patients access health services, they are often left feeling the cultural dissonance between Western biomedical health care models and traditional holistic approaches used in Indigenous communities (Davy et al., 2016; Kerrigan et al., 2021).

National guidelines for the diagnosis and management of these disease processes have been implemented (Remond et al., 2013; RHD Australia 2020) with the goal of eventual eradication. One such strategy implemented by the Australian government is the Rheumatic Fever Strategy (Quinn et al., 2019). Commencing in 2009, the strategy was a partnership between the federal government and the state governments of Western Australia, South Australia, Queensland, and the Northern territory—states and territories where ARF and RHD are notifiable diseases (RHD Australia, 2020)—with the aim of improving the detection, management, and monitoring of RHD and ARF. This involved better delivery of secondary prophylaxis (Australian Government Department of Health, 2017; Quinn et al., 2019), and maintenance of state registries, recall systems, and data collection (Australian Government Department of Health, 2017).

Secondary prophylaxis consists of benzathine benzylpenicillin injections given intramuscularly every 21–28 days (RHD Australia, 2020). The duration of secondary prophylaxis is dependent on several factors including age of patient, severity of disease, and date of most recent episode of rheumatic fever (Al-Jazairi et al., 2017). Traditionally, secondary prophylaxis was recommended to patients for a minimum of 10 years, or until the age of 21, whichever is longer (Ralph et al., 2021). However, the most recent guidelines published in 2020 changed this recommendation, so for those without cardiac involvement, secondary prophylaxis is recommended for a minimum of 5 years or until the age of 21, whichever is longer (Ralph et al., 2021; RHD Australia, 2020). Intramuscular injections every 21–28 days are the preferred first-line option (RHD Australia, 2020). Oral tablets taken twice a day are a second-line alternative if intramuscular injections are not possible (RHD Australia, 2020). This antibiotic strategy is the only one shown to decrease the reoccurrence of *A streptococcus* infections, slow the progression from ARF to RHD, and be cost effective (RHD Australia, 2020). However, oral penicillin has been shown to be less effective than their intramuscular counterparts for the prevention of *A streptococcus* infections and ARF recurrence (Ralph et al., 2021).

Patient adherence is summarized by the World Health Organization as the degree to which an individual’s actions or behavior correspond to that recommended by the health care practitioner (Engelman et al., 2016). Patient access to secondary prophylaxis for ARF is usually presented as a percentage of injections received to that prescribed (AIHW, 2017). Secondary prophylaxis continues to be a problem with ARF or RHD, with only 37%, who were overwhelmingly First Nations people, receiving at least 80% of the required doses in 2019 (AIHW, 2021). While 100% adherence to injections is considered the gold standard for secondary prophylaxis treatment, receiving 80% of scheduled doses is an indicator of adherence at a population level (AIHW, 2021). Rates of patients accessing secondary prophylaxis still remain low in Australia, with the Australian Medical Association calling it a “national failure” in 2018 (Liaw et al., 2019). There are several aspects that influence patients accessing treatment, and this integrative review aims to explore the barriers to secondary prophylaxis among First Nations people with ARF in Australia.

Cannon et al. (2019) predicted that the medical costs of caring for the 3,420 active cases, as of 2016, would be approximately AU$26.7 million. The analysis also projected another 10,211 cases by 2031 if current progression continues, amassing a medical cost of AU$317 million (Cannon et al., 2019). This is an avoidable cost if changes are made to alter the incidence, prevalence, and progression of ARF and RHD (Cannon et al., 2019). While this projection is not specific to secondary prophylaxis only, it does show the significant cost this preventable disease could have if no change occurs.

This prompts the topic and question of this review. The question aimed to be answered within this review is “What are the barriers to secondary prophylaxis of acute rheumatic fever in Australia’s First Nations people?” An integrative review was chosen as it allows for a thematic analysis of the literature to be critiqued and summarized (Christmals & Gross, 2017; Noble & Smith, 2018).

## Method

A population/concept/context framework (Archibald et al., 2016) was used to identify the components of this integrative review topic. The population was First Nations people in Australia with Rheumatic Fever. First Nations people were chosen due to the relatively high prevalence of ARF among this demographic compared with other Australians (Chamberlain-Salaun et al., 2016; Quinn et al., 2019). The concept of this review was secondary prophylaxis of ARF, with the context being patient adherence. Secondary prevention encompasses a vast number of strategies and is a complex and diverse subject. Therefore, secondary prophylaxis was chosen to limit the concept to a single component of secondary prevention, thereby allowing for a greater overview. Due to the low uptake of secondary prophylaxis among First Nations people, with less than 20% receiving their required doses (AIHW, 2021), barriers/adherence was chosen as the context.

Within this integrative review, a systematic search strategy was used to identify research articles that may pertain to the review topic (Noble & Smith, 2018). Databases were selected that covered many aspects of health care, including the nursing, medical, and public health perspectives. The databases searched were PubMed, Cumulative Index to Nursing and Allied Health Literature (CINAHL), ProQuest, and Wiley Online Library. Search terms used were “Acute Rheumatic Fever” AND “Secondary Prophylaxis” OR “Penicillin” AND “Aboriginal” OR “Indigenous Australians” AND “Barrier” OR “Adherence.” Please note that the terms “Indigenous Australians” and “Aboriginal” have been, and currently remain, the terminology most often found in the existing literature, with “First Nations” emerging as a preferred term.

**Figure 1.**
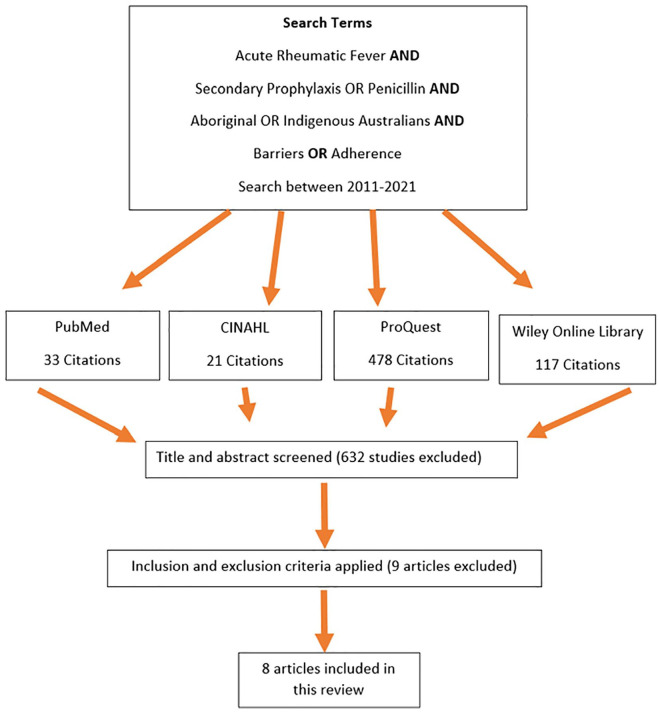
Articles Retrieved and Retained.

A previous systematic review of adherence to secondary prophylaxis in the global population, 1994–2014, was published in 2017 (Kevat et al., 2017). To ensure an adequate number of research articles were included without unnecessary overlap between reviews, a 10-year limitation was set of 2011–2021. A total of 649 citations were retrieved from the four databases. After the primary author screened the titles and abstracts, a total of 17 research studies remained. After considering inclusion and exclusion criteria (Table 1) and critically appraising the articles (Supplemental Appendix 1) using the appropriate Joanna Briggs Institute (JBI, 2021) appraisal tools to match the study type, eight articles remained and were included in this review. A flowchart of the full search is shown below in Figure 1.

**Table 1.** Inclusion and Exclusion Criteria for Screening Articles.

Inclusion criteria	Exclusion criteria
Primary research articles with full text available	Abstract-only articles
Articles published between 2011 and 2021	Articles not relating to Australian population research articles
Research pertaining to Indigenous Australians/First Nations People	Gray literature (non-peer-reviewed studies)
Rheumatic fever patients	Not relevant to research topic
	Unable to translate to English

## Synthesis of Findings

From the eight articles included in this review, six common themes were identified as barriers to secondary prophylaxis in ARF. The grouping of themes allows the data found within the research articles to be grouped and succinctly analyzed (Nowell et al., 2017). Thematic analysis was undertaken through a variation of the framework produced by Braun and Clarke (2006). In line with step one of Braun and Clarke (2006), the studies were read to better familiarize the reader with the topic. A mind map was used to complete the next three steps of the framework, to search, review, and define themes before the final step of writing up the review (Braun & Clarke, 2006). A total of six themes were identified within the research articles (Table 2), with one article discussing all the identified themes (Chamberlain-Salaun et al., 2016). The article summary table is found in Supplemental Appendix 2.

**Table 2.** Theme Summary Table.

Theme	Number of studies discussing theme	Studies pertaining to this theme
Databases and Recall Systems	4	Chamberlain-Salaun et al. (2016) Katzenellenbogen et al. (2020) Quinn et al. (2019) Ralph et al. (2013)
Patient Characteristics and Family Support	4	Chamberlain-Salaun et al. (2016) de Dassel et al. (2018) Kevat et al. (2021) Ralph et al. (2018)
Service Delivery Location and Site	3	Chamberlain-Salaun et al. (2016) de Dassel et al. (2018) Ralph et al. (2013)
Pain of Injection	2	Chamberlain-Salaun et al. (2016) Mitchell et al. (2018)
Education	3	Chamberlain-Salaun et al. (2016) Katzenellenbogen et al. (2020) Ralph et al. (2018)
Patient–Clinician Relationship	2	Chamberlain-Salaun et al. (2016) Mitchell et al. (2018)

### Theme 1: Databases and Recall Systems

Multiple databases and recall systems were found to be used for the service delivery of secondary prophylaxis. Chamberlain-Salaun et al. (2016) and Katzenellenbogen et al. (2020) highlighted the complex and multiple systems that clinicians need to engage with to capture and record data at both the local level to provide timely treatment, and the state-wide level as mandated by state legislation. This duplication of databases can affect the accuracy of the data recorded. In one example from Queensland, reported data needed to be entered into the RHD Queensland system and then cross-checked with recall lists on regional databases before local systems were updated for staff access (Chamberlain-Salaun et al., 2016). This was undertaken to ensure timely injection, but such duplication can lead to mistakes which can be a barrier to timely secondary prophylaxis administration (Ralph et al., 2013). The double and triple handling of this data can cause errors to occur in data collection (Health Policy Analysis, 2017; Katzenellenbogen et al., 2020), which in turn can also be a barrier to patients receiving secondary prophylaxis. Streamlining the process would improve the delivery of secondary prophylaxis (Ralph et al., 2013). Among patients receiving less than 80% of their prescribed injections, two studies found that patient recall systems using text message reminders and improved scheduling increased secondary prophylaxis (Chamberlain-Salaun et al., 2016; Ralph et al., 2013).

### Theme 2: Patient Characteristics and Family Support

There are a number of patient and family support characteristics that can influence patients receiving secondary prophylaxis (de Dassel et al., 2018; Ralph et al., 2018). Age was a significant influence, with two studies finding that adolescents received less secondary prophylaxis than their younger counterparts (Chamberlain-Salaun et al., 2016; de Dassel et al., 2018). Similarly, de Dassel et al. (2018) found that 30% of children received treatment compared with only 18% of adolescents. It is proposed that younger children have the support of a parent or carer to facilitate visits to clinics for injections (de Dassel et al., 2018), while adolescents are more independent but do not understand the consequences of not receiving regular secondary prophylaxis (Chamberlain-Salaun et al., 2016).

Kevat et al. (2021) reported that the strongest variable affecting pediatric patients receiving secondary prophylaxis was their family network. This was similarly reported by Chamberlain-Salaun et al. (2016) who found family support to be an enabling factor for seeking treatment, while Ralph et al. (2013) concluded that family meetings were a significant intervention for those receiving less than 80% of their prescribed secondary prophylaxis. In contrast, characteristics such as negative life experiences with hazardous use of alcohol and previous experience of assault were significant barriers to receiving secondary prophylaxis (de Dassel et al., 2018).

### Theme Three: Service Delivery Location and Site

Service delivery location and site were identified as barriers to secondary prophylaxis (Chamberlain-Salaun et al., 2016; de Dassel et al., 2018; Ralph et al., 2013). The majority of patients receiving their secondary prophylaxis received their injections in primary health care settings (Chamberlain-Salaun et al., 2016; RHD Australia, 2020). However, variability of service exists depending on clinic location and clinic practices. Clinicians, patients, and carers highlighted that traditional delivery in primary settings may actually be a barrier to treatment (Chamberlain-Salaun et al., 2016; Ralph et al., 2016). A clinician who had previously worked in the Northern Territory noted that they would administer injections in the community, wherever was comfortable for the patient, and also reported that this approach was tolerated very well (Chamberlain-Salaun et al., 2016). The same study obtained patient and carer perspectives, and reported they would prefer injections to be administered in a more relaxed environment such as their home (Chamberlain-Salaun et al., 2016). Along with the interview responses found in Chamberlain-Salaun et al. (2016), further evidence from the continuous quality improvement intervention shows that clinicians going into the community and providing home visits increased secondary prophylaxis rates (Ralph et al., 2013). While Ralph et al. (2013) did not discuss changing the location from their primary health care setting, they did explore the need to provide more effective outreach programs involving better communication between health services and enhanced access to transport arrangements ways of improving treatment rates in primary health care settings included in their study. In a study by de Dassel et al. (2018), it was found that people living in small communities with one health care center for all their medical needs made treatment easier for patients to access. In contrast, people living in urban areas, where clients may need to seek out specific clinics which may be more geographically dispersed, found this to be a barrier to treatment in contrast to the one health care center (de Dassel et al., 2018).

### Theme 4: Pain of Injection

Secondary prophylaxis in the form of benzathine penicillin injections is considered to be painful due to the viscosity of the solution as well as the total volume that needs to be injected (Mitchell et al., 2018). However, there are a number of components other than solution viscosity and volume that affects the perception of pain, which contributes as a barrier to secondary prophylaxis. For patients, the inconsistency in pain reduction strategies was a barrier, as well as perceived side effects such as malaise, lethargy, prolonged site pain, and fever (Mitchell et al., 2018). Both studies that discussed the theme of pain (Chamberlain-Salaun et al., 2016; Mitchell et al., 2018) also highlighted how the negative experience of pain not only affected the patient but also the parents and carers of children receiving regular injections. For carers of children requiring regular injections, watching their child in distress was also distressing for them (Chamberlain-Salaun et al., 2016; Mitchell et al., 2018). The distress of watching a patient in pain was also a deterrent for the clinician, because of the discomfort of injections to the patient made clinicians reluctant to do so (Mitchell et al., 2018). Clinicians also noted that patients who experienced pain were less likely to return for subsequent appointments (Chamberlain-Salaun et al., 2016).

### Theme 5: Education

The lack of health education, particularly ARF- and RHD-specific education among patients, parents/carers, family members, and health care providers was a barrier to treatment. Three studies showed that a lack of education, information, and understanding of ARF and RHD can be significant barriers (Chamberlain-Salaun et al., 2016; Katzenellenbogen et al., 2020; Ralph et al., 2018). For patients, empowerment can be achieved by improving patient education, which expands patients’ capacity to make informed decisions and aids in self-care and care of children and family (Chamberlain-Salaun et al., 2016). The quality improvement research highlighted that one of the most important interventions needed was client education (Katzenellenbogen et al., 2020), which helped decrease feelings of helplessness for both the patient and their parents/carers (Ralph et al., 2018). The RHD Secondary Prophylaxis Trial also showed that a lack of understanding regarding the need for regular injections was a barrier to adherence (Ralph et al., 2018). For clinicians, a lack of education about ARF and RHD was seen as a barrier to their own professional practice (Chamberlain-Salaun et al., 2016). An inability to progress their own knowledge and practice resulted in clinicians not being able to educate patients on the consequences of not adhering to secondary prophylaxis, which in turn would be a barrier to secondary prophylaxis (Chamberlain-Salaun et al., 2016).

### Theme 6: Patient–Clinician Relationship

Two studies showed that adequate patient-clinician relationships result in therapeutic encounters that allow for equity in power between the patient and the health care provider, promote feelings of safety for the patient to express concerns, and require providers to demonstrate cultural sensitivity and take time to listen and facilitate the development of shared treatment goals. The lack of an adequate patient–clinician relationship can be a barrier to secondary prophylaxis (Chamberlain-Salaun et al., 2016; Mitchell et al., 2018). Both studies indicate that trust between patient and clinician promotes patient comfort and improves willingness to openly discuss difficult topics such as injection related pain management, and various care concerns (Chamberlain-Salaun et al., 2016; Mitchell et al., 2018). However, fostering this type of relationship was found to be difficult due to the high prevalence of staff turnover that occurs in remote communities, where the majority of patients live (Chamberlain-Salaun et al., 2016). Mitchell et al. (2018) specifically focused on how the relationship, or lack thereof, affected the link between perceptions of pain and treatment rates. Both studies found that trust between patient and clinician made patients more comfortable about discussing their concerns about care with their clinician and help facilitate open discussion on strategies to minimize pain (Chamberlain-Salaun et al., 2016; Mitchell et al., 2018). Despite this, for seeking treatment, Chamberlain-Salaun et al. (2016) found that not all patients believed the patient-clinician relationship was important.

## Discussion

Key findings were compiled in the integrative review. This review builds on the previous systematic review completed by Kevat and colleagues (2017). However, while Kevat et al. (2017) article had a global perspective, this review aims to identify and discuss barriers specific to the First Nations people of Australia. This discussion section summarizes the findings and offers additional insights on ways to reduce barriers to engaging in secondary ARF prophylactic treatment by First Nations communities. One of the main themes was the importance of streamlining databases and recall systems, leading to better efficiency (Health Policy Analysis, 2017). This in turn means that patients can spend more time with their clinicians, receive more education, build the patient–clinician relationship, and thus increase the use of secondary prophylaxis (Chamberlain-Salaun et al., 2016; Mitchell et al., 2018). Furthermore, removing duplicate systems creates less error with data entry and more consistency and accuracy of data (Chamberlain-Salaun et al., 2016) and allows for patients to move between health services with a full record of their care. However, it is acknowledged that the actual task of streamlining these systems is problematic due to the numerous levels of systems that exist from the individual clinic to state and federal databases (Remond et al., 2013). The transfer and transition of care for those going from pediatric to adult services can also pose a barrier because patients may transition from having one practitioner looking after all facets of a person’s health to involving several health care providers for each comorbidity (RHD Australia, 2020).

The multiple levels of government that exist within Australia means that each state could potentially determine what is needed in different ways, which is further emphasized by the fact that ARF and RHD are not notifiable diseases in all states (RHD Australia, 2020). Streamlining databases requires consensus between states as well as at the federal level, something that has been attempted previously within the Rheumatic Fever Strategy, but was unsuccessful (Health Policy Analysis, 2017). An evaluation of the Rheumatic Fever Strategy recommended that the reorganization of databases and systems from a state to a national system would overcome current issues such as limitations in access to clinicians (depending on the system used), ability to access real time data across states, and have consistency in operational definitions and data structures (Health Policy Analysis, 2017). Liaw et al. (2019) proposed using the current national immunization registry as the database to record ARF and RHD data. The registry would be cost-effective because it is already used nationally, clinicians are already familiar with it, meaning that minimal staff training would be required, and up-to-date information would be more available if people require care away from home (Liaw et al., 2019).

The second theme of patient characteristics and family support acknowledged both modifiable and nonmodifiable factors that can be barriers to secondary prophylaxis (de Dassel et al., 2018). As demonstrated by Kevat et al. (2021), care needs to be tailored to the individual and their family network. This can be facilitated by another theme noted in this review—the patient-clinician relationship—which is something that requires time to build (Chamberlain-Salaun et al., 2016). By developing this relationship, individual care is more likely to be tailored to the individual and their family, part of which includes whether alternative sites of care delivery are needed to facilitate better rates of treatment (Chamberlain-Salaun et al., 2016). However, the high rates of clinician turnover that exist in remote communities (Ralph et al., 2018) mean this relationship is often not able to be fostered. Continuity of care allows for better follow up with patients, a factor found to influence access to secondary prophylaxis (Jones et al., 2020; Quinn et al., 2019). Less turnover also means a more positive clinical experience and the ability to solve issues such as injection pain. Increased experience means clinicians can streamline their technique and become more knowledgeable on devices that can be used to decrease injection pain (Mitchell et al., 2018), and improve patient education through consolidation in subsequent visits (Chamberlain-Salaun et al., 2016).

Inviting First Nations Health Practitioners can facilitate the bond between community and health service (Ralph et al., 2013). First Nations Health Practitioners, nationally registered as Aboriginal and Torres Strait Islander Health Practitioners, are First Nations people who work collaboratively in a primary care setting to provide culturally safe, high-quality care to First Nations people and their communities (Queensland Government, 2021). Creating a symbiotic educational relationship between First Nations health practitioners from within the community with clinicians from outside the community can benefit both parties. First Nations practitioners will be sensitive to the needs and preferences of their community and can engage and educate clinicians from outside the community on ways to provide culturally safe care that empowers individuals and their families. A systematic review that evaluated the effectiveness of cardiovascular health programs in First Nations communities concluded that there was greater empowerment in the community when there was appropriate training and involvement of First Nations health care workers (Mbuzi et al., 2018). While RHD guidelines have engaged various First Nations organizations—such as the Australian Indigenous Doctors Association, the Congress of Aboriginal and Torres Strait Islander Nurses and Midwives, and the National Aboriginal and Torres Strait Islander Health Worker Association (RHD Australia, 2020), more needs to be done at the grassroots level with these organizations and communities to provide more culturally sensitive, safe, and aligned care (Jones et al., 2020; RHD Australia, 2020). Additional health care provider incentives need to be created that make working remotely more attractive, including encouragement to undertake undergraduate and postgraduate education (Liaw et al., 2019) to facilitate the transition to remote practice. However, only two of twenty medical schools in Australia currently include education on ARF and RHD in their undergraduate program (Liaw et al., 2019). Changes to both undergraduate and postgraduate health curriculums are needed if any meaningful progress is to be made to ensure culturally safe, sensitive, and aligned medical education is taught and provided to First Nations people (Jones et al., 2020; Liaw et al., 2019). This includes courses taught by First Nations people and cultural experts.

A fundamental part of education is communication. Many First Nations people living in rural and remote communities do not speak English as their first language (Kerrigan et al., 2021); therefore, for effective education and communication with patients, engagement with First Nations health care workers and interpreters may be needed to ensure that culturally safe and competent care is provided (Jones et al., 2020; Kerrigan et al., 2021; Mbuzi et al., 2018; Mitchell et al., 2018). A pilot study conducted in the Northern Territory found that by involving interpreters in the care of First Nations people, clinicians were able to provide more culturally sensitive care as well as give control back to the individual, making them feel more empowered in their care (Kerrigan et al., 2021). However, it is important to remember that due to the variation that occurs between First Nations individuals and communities, it cannot be a “one size fits all” mentality (Kang et al., 2021). This is a complex issue that requires cultural sensitivity and a greater understanding of First Nations individual needs. For this to occur, any culture of discrimination and disadvantage must be acknowledged and reparations (or corrective actions) must immediately be made (Kerrigan et al., 2021; Mayes, 2020).

## Implications for Care

The review highlighted that a number of things need to change to influence future practice. First, the streamlining of multiple systems and databases across states and territories into a national RHD register would allow for more up-to-date information and may help improve continuity of care when moving across regions.

Steps to improve continuity of care for patients would be helpful in building trust. This means reducing current high staff turnover rates so that a patient-clinician relationship can be better developed. There is also the need for flexibility in offering community-based or home-based prophylactic administration. It is also highlighted in the literature that better management of pain of injection is needed. Finally, emphasis must be made within undergraduate and graduate education, and for continuing professional development, on the importance of culturally appropriate and linguistically aligned care to First Nations communities (Liaw et al., 2019).

## Strengths and Limitations

The main strength of this article is that it addresses issues specific to Australia’s First Nations people and does not combine data with other minorities affected by ARF and RHD in Australia. In addition, this paper aims to advocate for the health of a vulnerable population who are socioeconomically disadvantaged.

A limitation of this paper is the lack of a First Nations consultant (although we attempted to secure such input, we were unable to achieve it). This review also has limitations due to the wide range of terminology possible when referring to Australian First Nations people. The literature, however, will often refer to terms such as “Aboriginal” and “Indigenous,” so we are confident that the search has captured the overwhelming majority of available terms. This review did not include a statistical meta-analysis, but it was decided that the low number of articles made such an exercise redundant. Finally, only primary peer-reviewed published research was used, meaning some pertinent research may have been missed.

## Conclusion

There is complex interplay between the six themes identified; therefore, further collaboration between community, health services, and government is needed to create strategies to improve care by decreasing barriers and enabling treatment. This review provides evidence to support change in clinical practice by implementing corrective strategies that address well-known barriers such as pain of injection, site of service delivery, and support systems. Of equal importance, greater emphasis is needed on the other themes discussed within this review such as the need to streamline systems; retention of clinicians; better education of staff to provide culturally sensitive, safe, and aligned care; and increasing the role of First Nations health care workers to provide care specific to ARF and RHD. Prioritization of First Nations community needs will promote progress in influencing health care systems transformation, improving prophylaxis administration and treatment compliance for ARF and RHD, and most importantly, advancing health equity and improving health outcomes for high risk or socially disadvantaged First Nations Australians.

## Supplemental Material

sj-docx-1-tcn-10.1177_10436596231191248 – Supplemental material for Secondary Prophylaxis Among First Nations People With Acute Rheumatic Fever in Australia: An Integrative Review Click here for additional data file. Supplemental material, sj-docx-1-tcn-10.1177_10436596231191248 for Secondary Prophylaxis Among First Nations People With Acute Rheumatic Fever in Australia: An Integrative Review by Kerissa Govender and Amanda Müller in Journal of Transcultural Nursing
