# Supplementary material for: Secondary Prophylaxis Among First Nations People With Acute Rheumatic Fever in Australia: An Integrative Review
Source: J Transcult Nurs. 2023 Aug 12;34(6):443–52. doi: 10.1177/10436596231191248 (PMC10637076; doi:10.1177/10436596231191248)
Supplement: sj-docx-1-tcn-10.1177_10436596231191248 – Supplemental material for Secondary Prophylaxis Among First Nations People With Acute Rheumatic Fever in Australia: An Integrative Review [file sj-docx-1-tcn-10.1177_10436596231191248.docx]

# **Appendix 2**

# **Article Summary Table**

(Note that original terminology such as ‘Indigenous’ and ‘Aboriginal’ is used here because this is representative of the literature itself)

| **Primary author, date, location, *title*** | **Study Aim** | **Participants** | **Study method** | **Study results pertinent to review** |
| --- | --- | --- | --- | --- |
| Chamberlain-Salaun et al. (2016)  Queensland.  ***Sharing Success -understanding barriers and enablers to secondary prophylaxis delivery for Rheumatic Fever and Rheumatic Heart Disease.*** | Gain understanding of enablers and barriers of adherence to secondary prophylaxis in Indigenous Australians with Acute Rheumatic Fever and/or Rheumatic Heart Disease. | 24 participants encompassing patients, carers, and health professionals | Qualitative descriptive study consisting of semi-structured face to face interviews with patients, their care givers, or health practitioners. | Six themes were identified that affected the patient, their carer (if applicable) and health professionals that administer the prophylaxis. Barriers to secondary prophylaxis within these themes included:  - Registration Systems and Databases: duplicate and complexity of databases and state registers.  - Injection pain: For the patient, the parent/guardian if patient is a minor, and clinician administering the injection.  - Access and transport to location of secondary prophylaxis administration: proximity to service delivery and/or transport options to get to appointments.  - Education to patients, their carers and health professionals: lack of understanding of the importance and complications of not receiving recommended injections from patient and/or carer perspective. For clinicians, the lack of education meant they were not able to educate patients, and some reported initially not understanding the disease process or complications either of not adhering to secondary prophylaxis.  - Responsibility for seeking treatment and timely follow ups: Onus on patient and/or carer to come up appointments or staff or follow up and remind patient/carer.  - The role the healthcare practitioner-patient relationship plays on adherence to secondary prophylaxis: lack of a trusting relationship between clinician-patient related with decreased adherence. |
| de Dassel et al. (2018)  Northern Territory.  ***How many doses make a difference? An Analysis of Secondary Prevention of Rheumatic Fever and Rheumatic Heart Disease.*** | Determine the association between adherence to secondary prophylaxis and clinical outcomes of people with Acute Rheumatic Fever or Rheumatic Heart Disease as well as factors that are associated with adherence. | People living in the Northern Territory with a history of Acute Rheumatic Fever or Rheumatic Heart Disease who have been prescribed secondary prophylaxis. | Retrospective cohort design investigated factors associated with adherence. Nested case-control and case-crossover designs were used for the association between adherence and clinical outcomes. | Increased adherence, all statistically significant with a P value of <0.05, was associated with:  - female compared to male (AOR: 1.21 with 95% CI [1.02-1.44], P value = 0.03)  - living outside urban areas compared to urban areas (AOR: 1.46 with 95% CI [1.13-1.88], P value = 0.004)  - obesity compared to those who are not (AOR: 1.71 with 95% CI [1.11-2.63], P value = 0.015)  Decreased adherence, all statistically significant with a P value of <0.05, was associated with:  - older patients  - less severe disease (AOR: 0.66 with 95% CI [0.53-0.81], P value = <0.001)  - increased time since initial diagnosis (AOR: 0.62 with 95% CI [0.52-0.75], P value = <0.001)  - hazardous use of alcohol (AOR: 0.72 with 95% CI [0.53-0.98], P value = 0.035)  - experience of assault (AOR: 0.62 with 95% CI [0.46-0.85], P value = 0.002) |
| Katzenellenbogen et al. (2020)  Northern Territory, Queensland, Western Australia, and South Australia.  ***Priorities for improved management of Acute Rheumatic Fever and Rheumatic Heart Disease: analysis of cross-sectional continuous quality improvement data in Aboriginal primary healthcare centres in Australia.*** | Determine the variation in quality of care across age groups, in patients with Acute Rheumatic Fever or Rheumatic Heart Disease, according to key indicators. Identify individual patient factors and health centre factors that affect quality of care. | 1081 patients (96% identified as Indigenous Australian) from 63 primary healthcare centres that participated in the Audit and Best Practice for Chronic Disease National Research Partnership Act. | Cross-sectional observation study of clinical audit data of patients with Acute Rheumatic Fever or Rheumatic Heart Disease. Generalised linear mixed-effects logistic regression was used to analyse quality of care determinants. | 85% of patients, under 50 years, were prescribed secondary prophylaxis but only 46.7% of patients received 80% of their injections indicating inadequacy of systems in place to facilitate prescription into action.  Older age was found to be a negative factor (compared to those under 15 years old) for  - adherence target of 80% injection adherence  - timely doctor review and echocardiogram  - client education  Variability between health centres made quality of care determinants difficult to determine. |
| Kevat et al. (2021)  Queensland.  ***Adherence rates and risk factors for suboptimal adherence to secondary prophylaxis for rheumatic fever.*** | Determine rates of secondary prophylaxis adherence in paediatric population and examine factors that affect suboptimal adherence. | 277 children (259 identified as Indigenous) were included with a combined total of 7374 registered administration of injections. | Retrospective analysis of data from the Rheumatic Heart Disease register of Queensland. A multi-level mixed model was used to explore various factors effect on having injections at timely intervals. | No children received the recommended 80% of injections within a 28-day interval.  Most children live in communities with a low index of  - socio-economic advantage and disadvantage  - education and occupation.  Strongest identifiable contributing factor to the variation in injections is elements specific to the individual child and/or their family network.  Gender, ethnicity, suburb, number of people per dwelling, ARIA class, index of education and occupation and index of socio-economic advantage and disadvantage did not influence adherence. |
| Mitchell et al. (2018)  Northern Territory.  ***Aboriginal children and penicillin injections for Rheumatic Fever: How much of a problem is injection pain?*** | Investigate the experiences of injection pain among Acute Rheumatic Fever patients, and the health practitioners practice and experience of administering the injections. | Patients between 5-35 years old were recruited from territory Rheumatic Heart Disease register (119 participants).  Clinicians working within the 10 sites were invited to participate (59 participants) | Patient interviews were conducted in an ethnographical study while clinician interviews were completed in the qualitative component of a mixed-method community randomised trial. | Six themes were identified as factors that influenced participants experience of receiving (patients) and administering (clinicians) penicillin injections, 3 of which were identified as barriers:  - Negative experiences of pain: From patient, carer and/or clinician.  - Perceived side effects of the injection: including, but not limited to malaise, fevers, and lethargy.  - Inconsistent use of pain reduction strategies. |
| Quinn et al. (2019)  Throughout Australia.  ***Clinic factors associated with better delivery of secondary prophylaxis in acute rheumatic fever management.*** | Develop a way to measure secondary prophylaxis performance and identify organisational level factors that are associated with improved secondary prophylaxis performance. | 496 patient records from 36 clinics. 96% of patients identified as Aboriginal or Torres Strait Islander. | Cross-sectional de-identified data from the Audit and Best Practice for Chronic Diseases National Research Partnership were accessed to identify performance factors using regression analysis. | There was no difference in 28-day versus monthly regimens in proportion of patients receiving at least 80% of their prescribed injections (27% of patients).  Clinic characteristics did not influence performance of delivering secondary prophylaxis (P value all >0.05):   - State or territory - Governance: community or government managed - Location: city/regional or remote - Accreditation - Estimated service population.   At a clinic level, the following were found to positively influence performance of delivering secondary prophylaxis (P value all <0.05):   - Team structure and function - Systematic approach to follow up. |
| Ralph, et al. (2018)  Northern Territory.  ***Improvement in rheumatic fever and rheumatic heart disease management and prevention using a health centre-based continuous quality improvement approach.*** | To grow a better understanding of the gap between best and actual practice in the prevention and management of Rheumatic Heart Disease, and to improve management in collaboration with remote Indigenous Australian health services with a structured systems approach. | Six primary healthcare clinics in remote and regional Northern Territory. Project committee included:  - Project investigators  - Health service managers  - Clinicians  - Staff of NT RHD Control Program  - Staff of RHD Australia | A continuous quality improvement was developed with key stakeholders. The project involved cyclical feedback from participants to revise and improve processes. Data entry, analysis and reporting through One21seventy web-based information system. | Aggregated data analysis showed the continuous quality improvement project positively impacted on the following indicators of the delivery of care:  - Patient receiving at least 40% of their prescribed injections  - Interventions to increase adherence in patients receiving less than 80% of their prescribed injections:  - Education regarding prevention  - Family meetings  - Action plan  - Recall services including home visits, written and text message reminders  - Improved scheduling of fourth weekly injections.  - Review by doctor within two years. |
| Ralph, et al. (2018)  Northern Territory.  ***Improving delivery of secondary prophylaxis for Rheumatic Heart Disease in a high-burden setting: Outcome of a stepped-wedge, community, randomised trial.*** | Test whether engaging staff and patients at primary care clinics with a health system intervention would improve delivery of secondary prophylaxis. | Healthcare centres (total of 30) requiring secondary prophylaxis. Total of 402 participants, all identified as Aboriginal. | Stepped-wedge (3-month steps), pragmatic, community, randomised trial with an open cohort design using mixed-methods evaluation. | There was no statistical improvement in of adherence rates between baseline phase (46%), intensive phase (41%) and maintenance phase (50%) of patients receiving at least 80% of prescribed injections).  No association between adherence to secondary prophylaxis and quantifiable characteristics of health care centres (patient numbers per site, staff turnover, number of action items completed) but there are case site-specific examples of increased adherence with influence of these factors. |

CI: Confidence Interval; AOR: Adjusted Odds Ratio
